# Chronic noise-exposure exacerbates insulin resistance and promotes the manifestations of the type 2 diabetes in a high-fat diet mouse model

**DOI:** 10.1371/journal.pone.0195411

**Published:** 2018-03-30

**Authors:** Lijie Liu, Yi Huang, Cong Fang, Hongyu Zhang, Jing Yang, Chuanying Xuan, Fanfan Wang, Haiying Lu, Shuangfeng Cao, Yongfang Wang, Shengwei Li, Jun Sha, Mingming Zha, Min Guo, Jian Wang

**Affiliations:** 1 Medical College, Southeast University, Nanjing, China; 2 Institute of Life Sciences, Southeast University, Nanjing, China; 3 School of Human Communication Disorder, Dalhousie University, Halifax, Nova Scotia, Canada; Universidade do Estado do Rio de Janeiro, BRAZIL

## Abstract

Epidemiological studies have revealed that noise exposure was associated with an increased risk of type 2 diabetes mellitus (T2DM). However, the exact nature of that association remains to be elucidated. The present study is designed to examine the effects of chronic noise exposure on the development of T2DM in combination with a high-fat-diet (HFD) in mice. Here we show that chronic noise exposure at 85 dB SPL (4 h /day, below the safety limit for occupational noise exposure) exaggerated multiple metabolic abnormalities induced by HFD in C57BL/6J male mice, including worsened glucose intolerance, insulin resistance, fasting hyperglycemia and dyslipidemia. Furthermore, noise exposure exhibited a paradoxical impact on fat accumulation and circulating levels of free fatty acid, indicating a potential stimulating effect of noise on lipolysis. These results provide first *in vivo* supporting evidence for the causative role of noise exposure in diabetogenesis and pinpoint a noise-associated increase in blood free fatty acid levels as a possible mediator accelerating the effect of noise on the development of insulin resistance and T2DM.

## Introduction

Type 2 diabetes mellitus is a serious and common chronic disease characterized by hyperglycemia and impaired carbohydrate, lipid, and protein metabolism. The worldwide prevalence and associated high morbidity and mortality rates make this disease undoubtedly one of the most challenging health problems of the 21st century[1]. Although its’ exact etiology remains unknown, accumulating evidence suggests that T2DM is a quintessential multifactorial disease associated with numerous environmental influences that interact with the genetic predisposition underlying this disease[2]. Identification of risk factors is one of the top priorities in research seeking methods to delay or even prevent the onset of T2DM in the general population.

Noise, one of the most widespread sources of environmental pollution, is expanding in scope and intensity commensurate with the rapid urbanization and industrialization of modern society. Recently, a population-based study reported that exposure to residential traffic noise was associated with a greater risk of diabetes and the association was stronger for higher-level exposure and slightly stronger for long-term exposure[3]. Previous studies from our group provided the first experimental evidence that noise exposure at the safety limit (95 dB SPL, 2 h/day) adversely influences glucose metabolism and leads to insulin resistance in a “dose-dependent” manner (longer noise exposure correlates with longer-lasting insulin resistance) in mice[4]. A more recent experimental study also demonstrated persistently decreased hepatic insulin sensitivity in rats exposed to chronic noise at 100 dB SPL (4 h/day)[5]. Since insulin resistance is one of the major pathogenic disturbances involved in the development of diabetes, these studies suggest a contributory role of noise exposure in increasing the risk of T2DM. However, no direct evidence has been offered to establish the potential causative relationship between noise exposure and the onset of T2DM thus far.

Here, we investigated the effect of noise exposure upon T2DM development in combination with a high-fat-diet (HFD) in the C57BL/6J mouse strain, which is a widely used diabetic animal model with low baseline occurrence of T2DM, but high susceptibility to the development of diet-induced T2DM[6, 7]. The noise exposure was made closer to what can be experienced in daily life: a chronic broadband noise at 85 dB SPL, 4 hours per day for 2 to 8 weeks. Our experimental results demonstrated that the noise exposure at such a moderate level exaggerated glucose intolerance, insulin resistance, fasting hyperglycemia and dyslipidemia induced in HFD fed mice.

## Materials and methods

### Animals

Seven-week-old male C57BL/6J mice were obtained from the Nanjing University Animal Laboratories, China (Certification No. SCXK(SU) 2015–0001). In total, 64 mice passed the Preyer reflex test (an easy estimate of auditory function[8]) were used in the study. The mice were housed in regular polycarbonate plastic cages, four mice per cage, with a 12 h light/dark cycle (lights on at 7 A.M.) and had free access to food and water. One week after arrival, the mice were randomly assigned into two groups and fed either a chow diet (CD) or high-fat diet (HFD). On a caloric basis, the chow diet (TP23403, Trophic Animal Feed High-Tech Co., Ltd. Nantong, China) consisted of 10% fat from lard, 14.1% protein, and 75.9% carbohydrate (total 14.64 kJ/g), whereas the high-fat diet (TP23400, Trophic Animal Feed High-Tech Co., Ltd. Nantong, China) consisted of 60% fat from lard, 25.9% carbohydrate, and 14.1% protein (total 20.92 kJ/g). Half of each group was exposed to a broadband noise at 85 dB SPL for 4 h per day (between 6:00 p.m. and 10:00 p.m.) for up to 8 successive weeks (named as CD-N and HFD-N groups, respectively), while the other half were not subjected to noise-exposure (named as CD-C and HFD-C groups, respectively). To assess the effects of treatment on glucose homeostasis, intraperitoneal glucose tolerance tests (IPGTT) and intraperitoneal insulin tolerance tests (IPITT) were carried out (separated by two days to allow for recovery) after 2 weeks (2W), 4 weeks (4W), and 8 weeks (8W) of treatment. To minimize the number of animals used, the harvesting of tissue and blood was only carried out at 2W and 8W. Animals were treated humanely and with regard for alleviation of suffering during experimental procedures (e.g., by the utilization of well-trained animal research personnel and the administration of anesthesia, etc.). All animal procedures were approved by the University Committee for Laboratory Animals of Southeast University, China.

### Noise exposure

Noise exposure was carried out as described previously[4]. Briefly, conscious and unrestrained mice placed in separate metal net cages were exposed to the noise generated by a System III processor from Tucker-Davis-Technologies (TDT, Alachua, FL, USA) after a 30-min acclimation to the noise-exposure setting. The acoustic spectrum of the sound produced by the speakers after power amplification was distributed primarily between 1 kHz and 20 kHz. The noise level was set up at 85 dB SPL and monitored during the exposure using a 1/4-inch microphone linked to a sound level meter (Larson Davis 824, Depew, NY, USA).

### Intraperitoneal glucose tolerance test (IPGTT)

After 2, 4, and 8 weeks of treatment, IPGTT was performed in 16 h overnight-fasted animals with D-glucose (2 mg/g body weight, i.p.) dissolved in 0.9% saline. Blood samples were obtained from the tail vein just prior to (0 min) and at 10, 30, 60 and 120 min after the glucose injection. The blood glucose level was determined using a Bayer Contour glucose monitor (Bayer HealthCare LLC, Whippany, NJ). Measurement of the serum insulin levels prior to (0 min) and at 30 min after glucose injection was carried out using an ELISA kit (Cat # EZRMi-13k, from Millipore, Billerica, MA, USA). The levels of blood glucose and serum insulin recorded during the IPGTT were used for evaluating glucose tolerance and insulin response to the glucose challenge, respectively. IPGTT results were expressed as both a time course of absolute blood glucose measurements and as the area under the curve (AUC_GTTglucose_). The blood glucose and serum insulin before glucose loading (0 min) were taken as fasting blood glucose level (FBG) and fasting serum insulin level (FSI). To estimate basal insulin sensitivity, a homeostasis model of insulin resistance (HOMA-IR) was calculated as [FBG (in mmol/L) × FSI (in microunits/mL)] / 22.5[9, 10]. The insulinogenic index (an estimate of insulin response to glucose challenge) was calculated by dividing the incremental area under the curve (iAUC) of insulin during the first 30 min of the IPGTT by the iAUC of glucose over the same period [iAUC_Insulin(0–30)_ / iAUC_Glucose(0–30)_][11, 12].

### Intraperitoneal insulin tolerance tests (IPITT)

For the IPITT, a dose of 0.75 U/kg of regular human insulin (Humulin) was injected intraperitoneally in 4-hour fasted mice. Blood samples were taken from the tail veins before (0 min) and at 15, 30, 60 and 120 min after insulin injection and the blood glucose concentrations were measured using the same glucometer as above. IPITT results were expressed as both a time course of absolute blood glucose measurements and as the area under the curve (AUC_ITT_). As a widely used ITT-derived index of *in vivo* systemic insulin sensitivity, the blood glucose reduction rate (*K*_ITT_) was calculated with the formula 0.693 × t_1/2_^−1^ administration [9, 13]. The blood glucose half-life (t_1/2_) was calculated from the slope of the blood glucose concentrations from 0 to 30 min after insulin administration [9, 13].

### Tissue harvest

Two batches of animals from each group were sacrificed for the tissue harvest after 2 and 8 weeks of treatment, respectively. The mice were decapitated rapidly by a well-trained technician with sharp scissors 20 min after the intraperitoneal injection of insulin (Humulin, 0.75 U/kg body weight). The gastrocnemius muscle and the liver were dissected and immediately frozen in liquid nitrogen for immunoblot analysis or immersed in 4% (vol./vol.) paraformaldehyde solution for immunohistochemistry. Inguinal and epididymal adipose deposits were carefully collected and weighed for the evaluation of the white adipose tissue (WAT) content.

### Immunohistochemistry and morphological analysis

Immunofluorescent staining and morphological analysis for GLUT4 in skeletal muscle were performed as described previously [4]. Specimens of the gastrocnemius muscles were fixed in 4% paraformaldehyde for 24 h and then soaked in 30% sucrose in PBS at 4°C overnight. Next, the samples were embedded in Optimal Cutting Temperature compound (OCT, Leica Instruments, United Kingdom). Five consecutive sections (approximately 100 μm apart) from each specimen were permeabilized with 0.01% Tween and blocked with 5% BSA for 1 h at 37°C and then incubated with anti-GLUT4 (ab33780; Abcam, Cambridge, UK) overnight at 4°C. After PBS washing, the sections were incubated with AlexaFluor 488-conjugated chicken anti-rabbit antibodies (A-21441, Molecular Probes, Eugene, OR) for 1 h at 37°C. The DNA fluorochrome 4', 6-diamidino-2-phenylindole (DAPI) was used to stain the nuclei. Images were obtained using an Olympus IX71 inverted fluorescence microscope (Tokyo, Japan) and were analyzed with ImageJ. The relative GLUT4 content in the muscle cells was calculated using the average intensity of GLUT4 immunofluorescence per cell after normalization to the background.

### Protein preparation and western blot analysis

As described previously [4], gastrocnemius muscle and the liver samples were processed by homogenizing in ice-cold RIPA buffer (Beyotime P0013C, China) supplemented with complete protease inhibitor cocktail (Roche, Germany) and PhosSTOP (Roche, Germany). The protein concentration of the supernatant obtained by centrifugation was determined with a BCA protein assay kit (Pierce Biotechnology, Rockford). The protein extracts (40 μg) for each preparation were separated by SDS-PAGE and analyzed by specific antibodies including anti-AKT (Cell Signaling Technology, Cat no. #4685, Beverly, MA, USA), anti-phospho-AKT Ser^473^ (Cell Signaling Technology, Cat no. #4058, Beverly, MA, USA), anti-IRS1 (Cell Signaling Technology, Cat no. #2382, Beverly, MA, USA), and anti-phospho-IRS1 (Ser^307^) (Cell Signaling Technology, Cat no. #2381, Beverly, MA, USA). After incubation with horseradish peroxidase-conjugated secondary antibodies, the immunoblots were visualized with an ECL Kit (WBKLS0050; Millipore, Billerica, MA, USA), and quantitated by densitometric analysis using ImageJ.

### Serum biochemical analysis

At 2 and 8 weeks post-treatment, in addition to blood glucose measurements during IPITT, 30 μl blood samples were taken before the insulin injection for the assessment of the blood lipid profile. Blood samples were collected from the tail vein by pricking the vein using a sterile needle. Following centrifugation at 4°C, the resulting serum was separated and stored at −80°C for later biochemical analysis. Serum free fatty acids (FFA), triglycerides (TG) and total cholesterol (TC) were determined using commercially-available assay kits purchased from Nanjing Jiancheng Bioengineering Institute (Nanjing, China) according to the manufacturer’s instructions and guidelines.

### Statistics

Data were analyzed using GraphPad Prism 5.0 and expressed as the means ± standard error (SE) and *P* < 0.05 was deemed significant. For differences in blood glucose levels or insulin levels during the IPGTT or IPITT, a repeated two-way ANOVA (time and treatment) was performed and significance determined using Bonferroni’s *post hoc* test. All other data were analyzed by a two-way ANOVA (with the factors of diet and noise) followed by Bonferroni’s *post hoc* tests. As Prism does not compare the HFD-N and CD-C group in *post hoc* tests following two-way ANOVA, the differences between groups were further determined by one-way ANOVA followed by Tukey’s *post hoc* tests.

## Results

### Effect of noise exposure on glucose tolerance and insulin response to glucose challenge in mice

**Fig 1:**
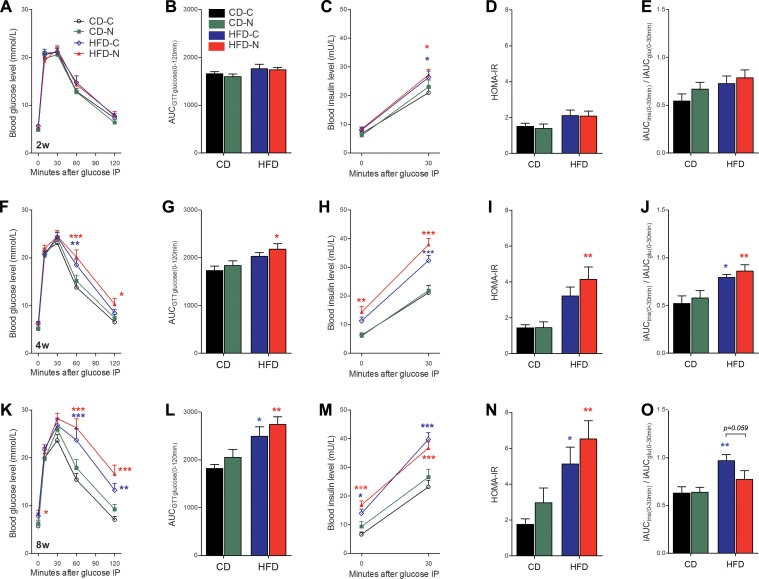
Effect of noise exposure on glucose tolerance and insulin response to glucose challenge in mice. (A, F, K) Blood glucose levels (BGL) recorded during the IPGTT in 16 h-fasted mice, performed after 2 weeks (A), 4weeks (F) and 8weeks (K) of treatment. (B, G, L) The corresponding results of A (B), F (G) and K (L) analyzed by area under the curve, i.e., AUC_GTTglucose_. (C, H, M) The serum insulin levels prior to (0 min) and 30 min after glucose injection (30SI) during the IPGTT corresponding to A (C), F (H) and K (M). (D, I, N) The homeostasis model of insulin resistance (HOMA-IR) derived from IPGTT corresponding to A (D), F (I) and K (N). (E, J, O) The iAUC_Ins(0–30)_/iAUC_Glu(0–30)_ derived from IPGTT corresponding to A (E), F (J) and K (O). The values are presented as the means ± SEM of 8 mice per group. The differently-colored asterisks relate to comparisons between the color-matched group and the CD-C group determined using Bonferroni’s *post hoc* test following a repeated two-way ANOVA (A, F, K, C, H, M) or Tukey's *post hoc* test following a one-way ANOVA (all other panels). The number of asterisks indicates the level of significance: *p < 0.05, **p < 0.01 and ***p < 0.001.

The baseline blood glucose concentrations prior to glucose administration (0 min, FBG) were comparable across 4 groups after 2 and 4 weeks of treatment (Fig 1A and 1F) and significantly elevated in the HFD-N group after 8 weeks of noise exposure (45.8% higher than CD-C group, Fig 1K). After glucose injection, the blood glucose level (BGL) of each group increased rapidly, peaking within 30 min, and then gradually decreasing. The curves largely overlapped between the two CD groups (CD-C and CD-N) after 2 weeks of noise exposure, and elevated slightly (but not significantly) in the recovery phase in the CD-N group after 4 and 8 weeks of noise exposure. The same trend was also illustrated by the measurement of AUC_GTTglucose_ (Fig 1B, 1G and 1L). This result suggests that noise-exposure alone did not significantly affect glucose tolerance. The curves measured from both HFD-C and HFD-N groups and the corresponding AUC_GTTglucose_ were also comparable to each other and to those from the two CD-fed groups after 2 weeks of the treatment (Fig 1A and 1B). After 4 and 8 weeks of treatment, both HFD groups exhibited higher BGL during the recovery phase and higher AUC_GTTglucose_, consistent with the overwhelming deleterious effect of the HFD (Fig 1F, 1G, 1K and 1L). Notably, although no significant difference was found between the two HFD groups in the values of BGL and AUC_GTTglucose_, only the HFD-N group exhibited significantly higher than in CD-C group BGL at 120 min post-glucose injection (BGL_120_, by 57.4%, *p* < 0.05) and AUC_GTTglucose_ (by 25.8%, *p* < 0.05) after 4 weeks of treatment (Fig 1F and 1G). A significant noise-exposure effect on BGL_120_ was evident after 8 weeks of treatment by a two-way ANOVA (*F* = 4.52, *p* = 0.042 for noise; *F* = 26.99, *p* < 0.001 for HFD). These results suggested that the noise exposure accelerated the deterioration of glucose tolerance induced by HFD.

The serum insulin level prior to and 30 min after glucose administration (FSI and 30SI) (Fig 1C, 1H and 1M) and HOMA-IR scores (Fig 1D, 1I and 1N) of CD-C and CD-N groups were all comparable after 2 and 4 weeks of treatment, but elevated slightly in the CD-N group after 8 weeks of treatment (FSI by 40.4% and 30SI by 14.5%). The FSI, SI30, HOMA-IR scores and the insulinogenic index [iAUC_Insulin(0–30)_ / iAUC_Glucose(0–30)_] of both HFD-C and HFD-N groups were generally increased throughout the entire experiment, indicating a development of insulin resistance and β-cell compensation in HFD-fed mice. No statistical difference between the HFD-C and HFD-N was seen at any of the time points. However, after 4 weeks of treatment, a significant difference in FSI (Fig 1H) and HOMR-IR (Fig 1I) was evident between HFD-N and CD-C (by a 127.1% and a 186.6% increase in HFD-N, respectively), but not between HFD-C and CD-C, suggesting a promoting effect of noise on the development of HFD induced insulin resistance in mice. Furthermore, there was a significant effect of noise exposure on FSI (a widely accepted surrogate of insulin resistance) as determined by two-way ANOVA for diet and noise exposure after 8 weeks of treatment (*F* = 4.30, 1, 28 DF, *p* = 0.0475). These results collectively indicate the detrimental effects of noise exposure on insulin activity. Interestingly, the HFD-N group exhibited a considerable lower value of iAUC_Insulin(0–30)_ / iAUC_Glucose(0–30)_ compared to the HFD-C group (by 30.4%, *p* = 0.059) after 8 weeks of treatment (Fig 1O), suggesting that a decline of β-cell compensation might have occurred in these animal after the long-term noise exposure.

### Effect of noise exposure on systemic insulin sensitivity in mice

**Fig 2:**
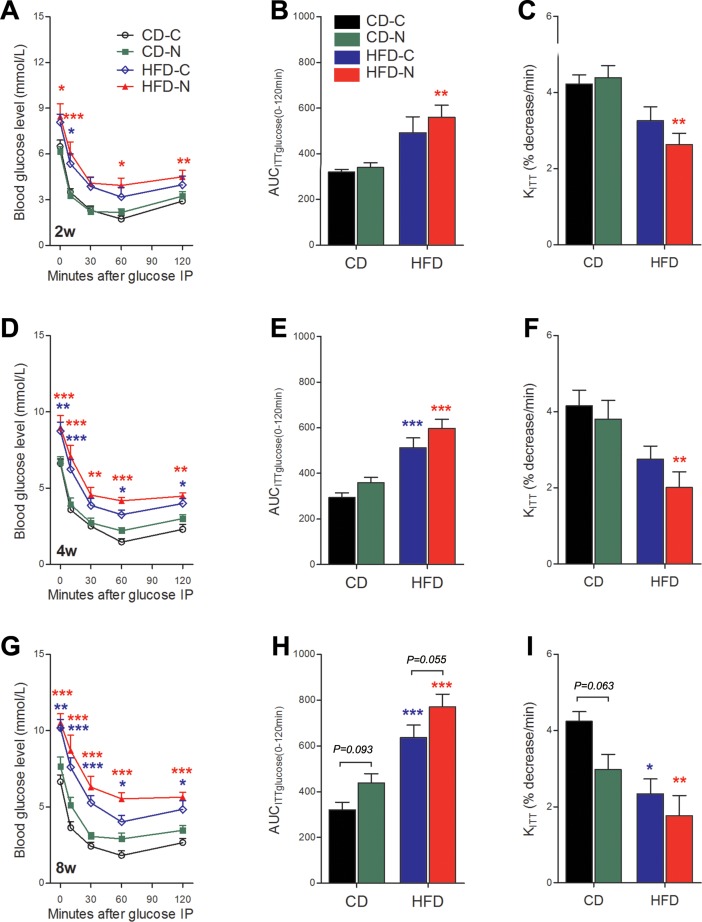
Effect of noise exposure on systemic insulin sensitivity in mice. (A, D, G) The IPITT blood glucose levels of 4 h-fasted mice performed after 2 weeks (A), 4weeks (D) and 8weeks (G) of treatment. (B, E, H) The corresponding results of A (B), D (E) and G (H) analyzed by area under the curve, i.e., AUC_ITTglucose_. (C, F, I) The plasma glucose disappearance rate (*K*_ITT_) derived from IPITT corresponding to A (C), D (F) and G (I). The values are presented as means ± SEM of 8 mice per group. The differently-colored asterisks relate to comparisons between the color-matched group and the CD-C group determined using Bonferroni’s *post hoc* test following a repeated two-way ANOVA (A, D, G) or Tukey's *post hoc* test following a one-way ANOVA (all other panels). The number of asterisks indicates the level of significance: *p < 0.05, **p < 0.01 and ***p < 0.001.

Compared to the CD-C groups, the CD-N groups showed a downward trend in *K*_ITT_ scores during the IPITT throughout the entire experiment (Fig 2). The decline of *K*_ITT_ was approaching although not reaching significance (by 29.8%, *P* = 0.063) after 8 weeks of treatments. Combined with the IPGTT results (Fig 1), these data suggest a slight, but progressively-declining insulin sensitivity in chronically noise-exposed CD-fed mice.

Both HFD-C and HFD-N groups exhibited a progressive decline in glucose response to insulin, as illustrated by increased BGLs, larger AUC_ITTglucose_ values and lower *K*_ITT_ scores in IPTTT throughout the experiment. This is consistent with the significant, progressively-deleterious effect of long-term HFD consumption on insulin sensitivity. Overall, the glucose response to insulin was worse in the HFD-N group compared to the HFD-C group, as indicated by the clear (barely fail to reach significant) difference (by 21.2%, *p* = 0.055) in the value of AUC_ITTglucose_ after 8 weeks of treatment (Fig 2H). The early significant increase of AUC_ITTglucose_ values (by 53.9%, *p* < 0.01) and significant decrease of *K*_ITT_ scores (by 22.8%, *p* < 0.01) in the HFD-N group after 2 weeks of treatment (Fig 2B and 2C) indicates that noise exposure hastened the development of insulin resistance in HFD-fed mice. The two-way ANOVA against diet and noise exposure revealed a significant effect of noise exposure on AUC_ITTglucose_ after 4 weeks of treatment (*F* = 5.11, 1, 28 DF, *p* = 0.0318) and 8 weeks of treatment (*F* = 7.00, 1, 28 DF, *p* = 0.0132), respectively, as well as on *K*_ITT_ score after 8 weeks of treatment (*F* = 5.06, 1, 28 DF, *p* = 0.0326) (Fig 2E, 2H and 2I). Together these data indicate a cumulative-dose-related exacerbative effect of noise exposure on the development of insulin resistance.

### Effect of noise exposure on insulin signaling pathways in skeletal muscle and liver

**Fig 3:**
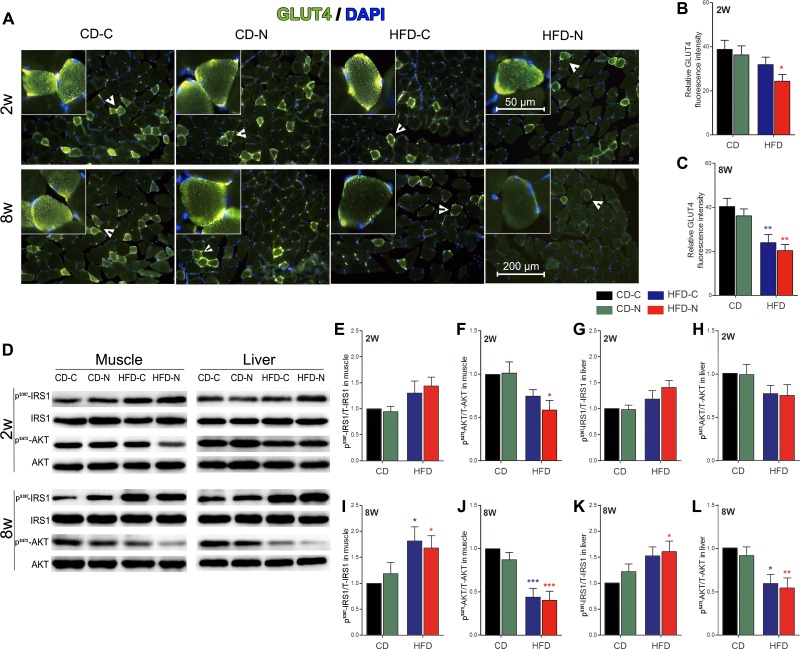
Effect of noise exposure on insulin signaling in the gastrocnemius muscle and liver. (A) Representative images of gastrocnemius sections stained for GLUT4 (green) and DAPI (blue). The insets show higher magnifications of GLUT4-enriched cells signified by arrowheads. All images were collected with a 20× objective. (B-C) The relative GLUT4 fluorescence intensity in gastrocnemius muscle cells 20 min after exogenous insulin injection after 2 weeks of treatment (B) and 8 weeks of treatment (C) normalized to background and compared across groups. (D) The levels of Ser^307^ phosphorylated IRS-1 (p^S307^-IRS1), total IRS1, Ser^473^ phosphorylated AKT (p^S473^-AKT) and total AKT detected by immunoblotting. (E-L) Phosphorylation levels of IRS1and AKT quantified and normalized to corresponding CD-C group. The average of each CD-C group was set to 1. The values are presented as the means ± SEM of 8 mice per group. *p < 0.05, **p < 0.01 and ***p < 0.001 indicate significance in the Tukey’s *post hoc* comparisons between each group and the CD-C after one-way ANOVA.

As illustrated by the immunohistochemical study in gastrocnemius sections (Fig 3A), twenty minutes after exogenous insulin injection, the GLUT4 signal of CD-C mice was predominantly localized to the cell membrane and was concentrated in a granular pattern in the periphery of the muscle cell. GLUT4 staining in the cell membrane and cytosolic fraction was notably weaker in the HFD-C and HFD-N mice after 8 weeks of treatment compared to the staining of CD-C group. Semi-quantitative analysis of relative GLUT4 fluorescence intensity showed a significant decrease in both HFD-C (by 40.6%, *p* < 0.01) and HFD-N (by 49.6%, *p* < 0.01) groups after 8 weeks of treatment (Fig 3C). Only the HFD-N group showed a significant decrease (by 37.3%, *p* < 0.05) in the GLUT4 signal after 2 weeks of treatment (Fig 3B). Western blot analysis of the skeletal muscle and the liver (Fig 3D–3L) demonstrated that there is no significant difference in phosphorylation levels of the insulin receptor substrate-1 (IRS-1) and protein kinase B (AKT) between the CD-N and CD-C groups after 2 weeks and 8 weeks of treatment. Compared to the CD-C group, both HFD-C and HFD-N groups exhibited significantly increased IRS-1 Ser^307^ phosphorylation and decreased AKT Ser^473^ phosphorylation in muscle and liver after 8 weeks of treatment (Fig 3D, 3I–3L). Combined with the GLUT4-immunofluorescence, these results exhibited a significant deleterious effect of HFD on insulin signaling. Although no significant difference was seen between the noise group and diet-matched control group, after 2 weeks of treatment, significant difference in GLUT4 (Fig 3B) and AKT Ser^473^ phosphorylation (Fig 3F) in skeletal muscle was only observed in the HFD-N (37.3% and 41.2% decline; *p* < 0.05) but not HFD-C group, providing further clues toward an promoting effect of noise on HFD induced insulin-signaling deterioration.

### Effect of noise exposure on bodyweight, body composition and blood lipid profiles in mice

**Fig 4:**
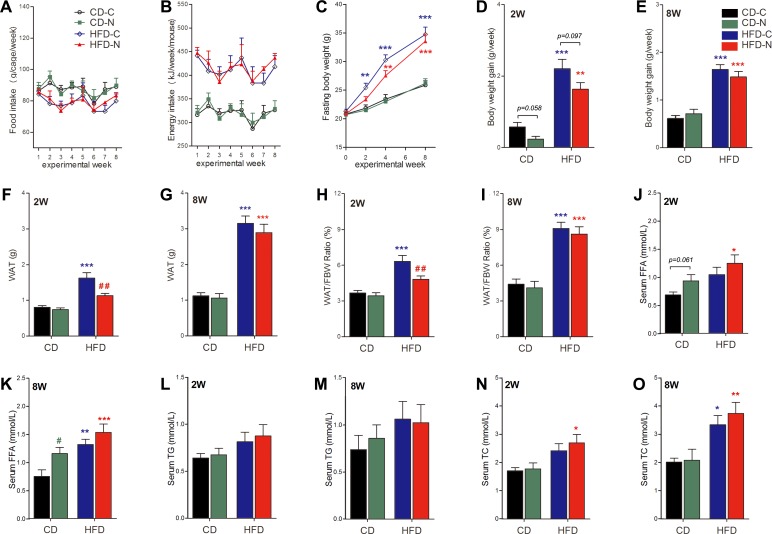
Effect of noise exposure on body composition and blood lipid profiles. (A) Food intake was measured on a per-cage basis once per week. The values are presented as the means ± SEM of 4 cages per group in first 2 weeks and 2 cages per group in following 6 weeks. (B) Energy intake calculated based on average weekly food consumption per mouse and dietary energy density. The values are presented as the means ± SEM of calculated energy intake per mouse from each cage. (C) The bodyweight of 16-hour fasted mouse was taken as fasting bodyweight (FBW). Values are presented as the means ± SEM of 16 (measured at experimental week 0, before the treatment) or 8 (measured before the IPGTT after 2, 4, and 8 weeks of treatment) mice per group. (D-E) Bodyweight gain of each group in the first 2 weeks (D) and throughout the 8-week treatment (E). (F-G) White adipose tissue (WAT) weight of each group after 2 weeks (F) and 8 weeks (G) of treatment. (H-I) The ratio of WAT over bodyweight after 2 weeks (H) and 8 weeks (I) of treatment. (J-K) Serum FFA concentrations of each group after 2 weeks (J) and 8 weeks of treatment (K). (L-M) Serum TG. (N-O) Serum TC. Values are presented as the means ± SEM of 8 mice per group. *p < 0.05, **p < 0.01 and ***p < 0.001 indicate the significance in the Tukey’s *post hoc* comparison between each group and the CD-C after a one-way ANOVA. #*p* < 0.05 and ##*p* < 0.01 indicate the significant difference between the noise group and diet-matched control group (CD-N vs CD-C and HFD-N vs HFD-C) determined using Bonferroni’s *post hoc* test following a two-way ANOVA with the factors of diet and noise.

The food intake of all groups was similar throughout the experiment (Fig 4A), but the average energy intake (calculated based on average weekly food consumption per mouse and dietary energy density) was much higher in HFD–fed mice (413.84 ± 5.28 kJ/week) than CD–fed mice (318.52 ± 3.37 kJ/week) (Fig 4B). The initial fasting bodyweight (FBW) was comparable between all groups (Fig 4C). Although no significant difference in FBW was shown between two CD groups at each time point, the bodyweight gain of the CD-N group in first two weeks was clearly lesser than that of the CD-C group (52.6%, *p* = 0.058) (Fig 4D). As expected, both HFD–fed groups exhibited significantly higher FBW at the end of the study. However, only the HFD-C group shown significantly higher than in CD-C group FBW after 2 weeks of treatment (by 16.4%, *p*<0.01). The HFD-C group also exhibited significantly higher bodyweight gain (Fig 4D and 4E, by 279.6% and 167.4%), greater WAT (Fig 4F and 4G, by 102.1% and 180.5%) and an increased ratio of WAT to FBW (Fig 4H and 4I, by 72.3% and 106.1%), as observed after 2 and 8 weeks of treatment, which is consistent with an overwhelming obesogenic effect of HFD consumption. However, the obesogenic effect of the HFD was transiently retarded in the HFD-N group, as indicated by the clear (although not significant) difference in the bodyweight gain (Fig 4D, by 30.0%, *p* = 0.097) and the significant difference in the value of WAT (Fig 4F, by 35.9%, *p*<0.01) and WAT/FBW ratio (Fig 4H, by 27.0%, *p*<0.01) after 2 weeks of treatment. The two-way ANOVA for noise and diet revealed a significant effect of noise exposure on FBW (*F* = 4.64, 1, 28 DF, *p* = 0.040), bodyweight gain (*F* = 6.69, 1, 28 DF, *p* = 0.0152), WAT (*F* = 9.79, 1, 28 DF, *p* = 0.0041) and WAT/FBW (*F* = 7.43, 1, 28 DF, *p* = 0.0109) after 2 weeks of treatment. Furthermore, significant interaction effects between noise and HFD on WAT (*F* = 5.82, 1, 28 DF, *p* = 0.0227) (Fig 4F) and considerable interaction effects between noise and HFD on WAT/FBW (*F* = 4.02, 1, 28 DF, *p* = 0.0546) (Fig 4H) were revealed by two-way ANOVAs. These results indicate a transiently significant effect of noise exposure attenuating weight gain and fat accumulation.

Analysis of serum lipid composition (Fig 4J–4O) revealed significantly increased FFA and TC concentrations in both HFD-C (FFA by 76.2%, *p*<0.01; TC by 65.7%, *p*<0.05) and HFD-N (FFA by 105.2%, *p*<0.001; TC by 85.4%, *p*<0.01) groups after 8 weeks of treatment (Fig 4K and 4O), illustrating the deleterious effect of HFD on lipid metabolism. Contrary to the diminished bodyweight gain and fat accumulation depicted above, the lipid profiles of noise-exposed animals were worse than diet-matched controls. Two-way ANOVA for noise and diet showed a significant effect of noise on FFA levels (*F* = 6.98, 1, 28 DF, *p* = 0.0133) after 8 weeks of treatment (Fig 4K). An obvious, although not significant (*F* = 3.64, 1, 28 DF, *p* = 0.0667) effect was also evident after 2 weeks of treatment (Fig 4J). These results indicate a synergistic effect of noise exposure and HFD on the development of dyslipidemia and also suggest a complex mechanism underlining the disturbing effect of noise on lipid metabolism.

## Discussion

Noise has long been realized can cause direct and cumulative adverse effects on both auditory and non-auditory systems [14]. With the rapid urbanization and industrialization of modern society, noise pollution is more severe and more pervasive in everyday life than ever before. In an over 10-year prospective epidemiological study conducted by Sørensen et al. in a Danish cohort, each 10 dB increase in average road traffic noise at the current residence was found to be associated with a statistically significant 11% increased risk of incident diabetes, increasing to 14% when road traffic noise was estimated for all the places an individual had lived in the previous 5 years[3]. Their study raising the concern for noise as a risker factor of diabetes, particularly so given the global epidemic of this disease and the plague-spreading pattern of noise pollution[15]. Two recent experimental studies from different labs separately demonstrated the development of insulin resistance in animals subjected to chronic noise at the level of 95 dB for 4 h/day (mice) or 100 dB for 2h/day (rat)[4, 5], suggested a contributory role of noise exposure in increasing the risk of T2DM. In this study, we investigated the effect of noise exposure upon various markers associated with T2DM development in combination with a HFD in the C57/BL6J mouse strain. The animals in present study were chronically exposed to broadband noises presented at 85dB for 4 h/day. Based upon the Occupational Safety and Health Administration (OSHA) 5 dB exchange rate, this noise level is equivalent to 80 dB for 8 h, or 79 dBA for 8 h (dBA is the sound pressure level when an "A" contour filter is used according to the sensitivity of the human ear). The permissible exposure limit on the workplace recommended by OSHA is 90 dBA for 8 h. Noise pollution in town is often louder than 70 dBA and the noise levels of heavy traffic can reach or exceed 80 dBA(32, 36). Therefore, exposure setting in this study is lower than the occupational safety allowance and is frequent in urban environments.

T2DM is a group of metabolic diseases resulting from a complex genetic and environment interactions in combination with other risk factors such as obesity and dietary composition[16]. The early stages of the disease are characterized by insulin resistance accompanied by compensatory increases in insulin secretion by pancreatic β-cells for the maintenance of euglycemia. Substantial and sustained insulin resistance culminates in β-cell exhaustion. Insufficient β-cell compensation leads to the hyperglycemia and the onset of overt diabetes[17]. Here, high BGL during IPGTT and IPITT, high FSI, 30SI, iAUC_Ins(0–30)_ /iAUC_Glu(0–30)_ and HOMA-IR derived from IPGTT as well as low *K*_ITT_ scores derived from IPITT displayed in the HFD-C mice after 8 weeks of treatment are consistent with the early stages of T2DM. While barely any significant difference in the values of these parameters between the HFD-C and HFD-N group was revealed, the significant change of these markers was generally observed at an earlier time point in HFD-N group than that of HFD-C group. Furthermore, after 8 weeks of treatment, an obvious higher than HFD-C AUC_ITTglucose_ was exhibited in HFD-N group (Fig 2H, barely fails to reach the level of significance, *p* = 0.055). Moreover, only the HFD-N group developed a significantly elevated FBG concomitant with a decreased insulinogenic index [iAUCI_nsulin(0–30)_ / iAUC_Glucose(0–30)_] at the end of this study. Although the CD-N group did not shown significantly impaired glucose and insulin tolerance, the two-way ANOVA for noise and diet revealed a significant deleterious effect of noise on AUC_ITTglucose_ and *K*_ITT_, two frequently used indices of insulin sensitivity, after 4 and 8 weeks of treatment (Figs 1 and 2). Collectively, these *in vivo* data suggest an exacerbating effect of noise exposure on the development of insulin resistance and T2DM.

Glucose disposal in skeletal muscle and suppression of glucose production in the liver are key mechanisms by which insulin controls glucose homeostasis[18]. In skeletal muscle, insulin stimulates glucose uptake by recruiting GLUT4 from intracellular sites to the plasma membrane, which activates a signaling cascade involving IRS-1 and AKT. The IRS/AKT pathway also mediates insulin signaling in the liver. Defects in these signaling pathways are considered the major pathogenic disturbances underlying the development and progression of T2DM[19]. Decreased GLUT4 expression and cell membrane localization, increased IRS-1 phosphorylation at Ser^307^ and decreased AKT phosphorylation at Ser^473^ after insulin stimulation have been well documented in insulin resistance[20]. In this study, although no statistically significant difference between the noise exposed group and diet-matched control group was observed, only the HFD-N group exhibited significant decreased GLUT4 signal and AKT Ser^473^ phosphorylation in skeletal muscle after 2-weeks of treatment, providing further clues at the tissue-level toward an accelerating effect of noise exposure on the diabetogenic action of the HFD. Future studies based on a more rigorous design (e.g., setting up of non-insulin injected control groups and more comprehensive assessment of the key components of insulin signaling pathway) will be worthful to get a more complete picture of the effect of noise exposure on the insulin sensitivity.

Although the precise mechanisms linking HFD and T2DM are still unclear, excessive energy intake and excess bodyweight are widely considered to be major contributors to the diabetogenic effect of long-term HFD consumption. In this study, the averaged energy intake was similar between groups fed with the same diet, but much higher in HFD–fed mice compared to CD–fed mice. HFD-C mice exhibited significantly more bodyweight gain and white fat accumulation after 2 weeks of treatment, preceding the occurrence of significant glucose intolerance, insulin resistance and dyslipidemia. However, the obesogenic effect of HFD was retarded by the noise exposure. Intriguingly, the diminished obesogenic effect of HFD in the HFD-N mice was accompanied with worse dyslipidemia indicated by higher serum FFA and TC levels (Fig 4J–4O). Two-way ANOVA for noise and diet demonstrated a significant repressive effect of noise on bodyweight gain (Fig 4D) and fat deposition (Fig 4F and 4H) and a significant enhancing effect of noise on circulating FFA (Fig 4J and 4K).

Adipose tissue plays a dynamic and critical role in fueling metabolism. Chylomicron triglyceride-fatty acids derived from dietary fat that are not immediately oxidized, are stored primarily in adipose tissue. In turn, adipose tissue can be mobilized to release FFA into the vasculature for use by other organs as an energy supply[21]. Insulin plays an essential role in the control of lipid metabolism. Insulin not only facilitates the entry of glucose into adipocytes providing more material for triglyceride synthesis within the adipocyte, but also inhibits the breakdown of fat in adipose tissue by inhibiting the intracellular lipase that hydrolyzes triglycerides to release fatty acids. By these mechanisms, insulin is involved in adipose tissue expansion[22]. However, the ability of insulin to inhibit lipolysis and reduce the plasma FFA concentration is markedly impaired in insulin resistance. Dyslipidemia characterized by an increase in circulating FFA is one of the major abnormalities in the lipid profile of T2DM. As noted above, it is reasonable to interpret the significantly higher weight gain in HFD-C after 2 weeks of treatment and the dyslipidemia occurring after 8 weeks of treatment as metabolic biomarkers of the gradual progression of T2DM induced by HFD.

The causal links between elevated circulating FFA and insulin resistance/T2DM are complex and controversial. A large amount of evidence has demonstrated that sustained elevated plasma FFA can cause/aggravate insulin resistance[11, 23] and β-cell degradation[12, 24], the main pathogenic disturbances responsible for the development of T2DM. Thus, FFA has been proposed as an important mediator between many risk factors and diabetes[19, 25]. The considerably early elevation of blood FFA might serve as a contributing factor to augment the effects of noise exposure on the progress of insulin resistance and T2DM in this study.

Since lipolysis is the major source of circulating FFA, fine regulation of this process is crucial for the maintenance of body energy homeostasis, as well as for the prevention of metabolic diseases[26]. Excessive lipolysis may lead to higher circulating FFA and thus contribute to the development of insulin resistance and T2DM. Consistent with this view, Wu et al. reported that nicotine increases lipolysis, which results in bodyweight reduction and elevated levels of circulating FFA, thus causing insulin resistance[27]. Uchida et al demonstrated that increased lipolysis and blood FFA contribute to insulin resistance induced by stress from chronic restraint [28]. In this study, we observed reduced bodyweight gain and adipose tissue weight and elevated FFA levels in the noise exposed group compared to the diet matched control group. It is plausible that an inverse trend between white fat (especially WAT/FBW) and blood FFA levels in noise-exposed animals indicates upregulated adipose tissue lipolysis, which can help explain the paradoxical observations of lower body weight and poor glycemic control in noise-exposed mice. Additional and more comprehensive methods (e.g., quantification of mRNA/protein levels of lipases and regulatory proteins in the adipose tissues, manipulation of lipolysis) will be needed in future studies to clarify the cause of elevated circulating FFA observed in noise exposed animals as well as the role of elevated circulating FFA regarding the noise related increased risk of T2DM.

Noise has long been classified as a nonspecific environmental stressor. Multiple observational and experimental studies in human and animal subjects have indicated that noise exposure is associated with arousals of the autonomic nervous system and endocrine system [4, 5, 29–32]. Elevated circulating cortisol and catecholamine levels in the stress response have been demonstrated to lead to insulin resistance and the development of T2DM[33]. In our previous study, in addition to the impaired insulin sensitivity, a significant increase in serum corticosterone level was observed in noise-exposed mice[4]. Similarly, Cui et al. reported that chronic noise exposure induced a persistent increase in blood corticosterone level as well as glucose dysregulation in rats[5]. More recently, another experimental study also reported increases of glucose and cortisol serum levels in mice after 30 days noise exposure [34]. These separate studies provided consistent evidence pointing to the possibility that the stress effect is (at least a part of) the rationale behind the diabetogenic effects of noise. Stress hormones are generally considered as catabolic hormones that mobilize energy stores to prepare the body for the fight or flight response to stressors. Increased circulating FFA resulting from stress hormone-induced lipolysis has been suggested to be an important causative link between the stress response, insulin resistance and T2DM[28, 35]. Consistent with previous animal studies focused on the effect of chronic noise on body weight [36, 37], noise exposed mice in present study displayed decreased body weight gain compared with their diet-matched counterparts. As no significant differences in food consumption were found between the diet-matched groups, the decreased body weight gain as well as increased circulating FFA observed in noise exposed mice might be interpreted as catabolic consequences of noise stress. No determination of the stress response and limited evidence regarding the upregulation of lipolysis by noise exposure are significant limitations of this study. Also, we currently cannot answer why the difference in the body weight between noise-exposed and diet-matched control group was much greater in HFD-fed mice than in CD-fed mice (Fig 4C). The detection of stress-associated markers as well as the evaluation of metabolic activity should be emphasized in future study to provide solid information about mechanisms underlying the potential diabetogenic effects of noise.

Taken together, we provide what we believe is the first demonstration of such chronic moderate noise exposure promoting the development of insulin resistance in C57BL/6 male mice. Furthermore, and more importantly, noise exposure accelerated the development and progress of T2DM in HFD-fed mice. Compare with the HFD, a well-known major diabetogenic environmental factor, noise exposure only exhibited small propelling effect in many of the parameters assessed in this study. In clinical practice, the effects of noise at mild intensities might be so gradual and insidious that the adverse health effects of such noise may have been ignored. However, the adverse effect can accumulate and even lead to severe diseases[32]. As the onset and the development of T2DM involves complex interactions between multiple genetic and environmental factors, it’s difficult for now to surmise how much these noised-related small changes will promote the development of T2DM. However, considering the alarming increase in the prevalence of T2DM[1] and noise pollution[14, 38], as well as the increasing incidence of obesity due to excess food intake, our results are important to raise the awareness about reducing environmental noise exposure as and to better manage interventions and strategies for improvement of public health.

## References

[1] Zimmet PZ, Magliano DJ, Herman WH, Shaw JE. Diabetes: a 21st century challenge. The lancet Diabetes & endocrinology. 2014;2(1):56–64. Epub 2014/03/14. doi: 10.1016/S2213-8587(13)70112-8 . 24622669 10.1016/S2213-8587(13)70112-8

[2] Mensink M. Lifestyle intervention, glucose tolerance, and risk of developing type 2 diabetes mellitus. Metabolic syndrome and related disorders. 2005;3(1):26–34. Epub 2008/03/29. doi: 10.1089/met.2005.3.26 . 18370707 10.1089/met.2005.3.26

[3] Sorensen M, Andersen ZJ, Nordsborg RB, Becker T, Tjonneland A, Overvad K, et al Long-term exposure to road traffic noise and incident diabetes: a cohort study. Environmental health perspectives. 2013;121(2):217–22. Epub 2012/12/12. doi: 10.1289/ehp.1205503 ; PubMed Central PMCID: PMC3569689. 23229017 10.1289/ehp.1205503 PMC3569689

[4] Liu L, Wang F, Lu H, Cao S, Du Z, Wang Y, et al Effects of Noise Exposure on Systemic and Tissue-Level Markers of Glucose Homeostasis and Insulin Resistance in Male Mice. Environmental health perspectives. 2016 Epub 2016/04/30. doi: 10.1289/EHP162 . 27128844 10.1289/EHP162 PMC5010391

[5] Cui B, Gai Z, She X, Wang R, Xi Z. Effects of chronic noise on glucose metabolism and gut microbiota-host inflammatory homeostasis in rats. Scientific reports. 2016;6:36693 doi: 10.1038/srep36693 ; PubMed Central PMCID: PMC5095650. 27811997 10.1038/srep36693 PMC5095650

[6] Paul DS, Hernandez-Zavala A, Walton FS, Adair BM, Dedina J, Matousek T, et al Examination of the effects of arsenic on glucose homeostasis in cell culture and animal studies: development of a mouse model for arsenic-induced diabetes. Toxicology and applied pharmacology. 2007;222(3):305–14. Epub 2007/03/06. doi: 10.1016/j.taap.2007.01.010 ; PubMed Central PMCID: PMC2680915. 17336358 10.1016/j.taap.2007.01.010 PMC2680915

[7] Winzell MS, Ahren B. The high-fat diet-fed mouse: a model for studying mechanisms and treatment of impaired glucose tolerance and type 2 diabetes. Diabetes. 2004;53 Suppl 3:S215–9. Epub 2004/11/25. . 15561913 10.2337/diabetes.53.suppl_3.s215

[8] Valente MM, Bortolotto V, Cuccurazzu B, Ubezio F, Meneghini V, Francese MT, et al alpha2delta ligands act as positive modulators of adult hippocampal neurogenesis and prevent depression-like behavior induced by chronic restraint stress. Molecular pharmacology. 2012;82(2):271–80. doi: 10.1124/mol.112.077636 . 22572885 10.1124/mol.112.077636

[9] Kiechl S, Wittmann J, Giaccari A, Knoflach M, Willeit P, Bozec A, et al Blockade of receptor activator of nuclear factor-kappaB (RANKL) signaling improves hepatic insulin resistance and prevents development of diabetes mellitus. Nature medicine. 2013;19(3):358–63. Epub 2013/02/12. doi: 10.1038/nm.3084 . 23396210 10.1038/nm.3084

[10] Andrikopoulos S, Blair AR, Deluca N, Fam BC, Proietto J. Evaluating the glucose tolerance test in mice. American journal of physiology Endocrinology and metabolism. 2008;295(6):E1323–32. Epub 2008/09/25. doi: 10.1152/ajpendo.90617.2008 . 18812462 10.1152/ajpendo.90617.2008

[11] Herzberg-Schafer SA, Staiger H, Heni M, Ketterer C, Guthoff M, Kantartzis K, et al Evaluation of fasting state-/oral glucose tolerance test-derived measures of insulin release for the detection of genetically impaired beta-cell function. PloS one. 2010;5(12):e14194 doi: 10.1371/journal.pone.0014194 ; PubMed Central PMCID: PMC2996282. 21152029 10.1371/journal.pone.0014194 PMC2996282

[12] Ahlkvist L, Brown K, Ahren B. Upregulated insulin secretion in insulin-resistant mice: evidence of increased islet GLP1 receptor levels and GPR119-activated GLP1 secretion. Endocrine connections. 2013;2(2):69–78. Epub 2013/06/20. doi: 10.1530/EC-12-0079 ; PubMed Central PMCID: PMC3680955. 23781322 10.1530/EC-12-0079 PMC3680955

[13] Yagi S, Chow C, Lieblich SE, Galea LA. Sex and strategy use matters for pattern separation, adult neurogenesis, and immediate early gene expression in the hippocampus. Hippocampus. 2016;26(1):87–101. doi: 10.1002/hipo.22493 . 26179150 10.1002/hipo.22493

[14] Basner M, Babisch W, Davis A, Brink M, Clark C, Janssen S, et al Auditory and non-auditory effects of noise on health. Lancet. 2014;383(9925):1325–32. Epub 2013/11/05. doi: 10.1016/S0140-6736(13)61613-X ; PubMed Central PMCID: PMC3988259. 24183105 10.1016/S0140-6736(13)61613-X PMC3988259

[15] Dzhambov AM. Long-term noise exposure and the risk for type 2 diabetes: a meta-analysis. Noise & health. 2015;17(74):23–33. doi: 10.4103/1463-1741.149571 ; PubMed Central PMCID: PMC4918642. 25599755 10.4103/1463-1741.149571 PMC4918642

[16] Cani PD, Bibiloni R, Knauf C, Waget A, Neyrinck AM, Delzenne NM, et al Changes in gut microbiota control metabolic endotoxemia-induced inflammation in high-fat diet-induced obesity and diabetes in mice. Diabetes. 2008;57(6):1470–81. doi: 10.2337/db07-1403 . 18305141 10.2337/db07-1403

[17] Tiganis T. Reactive oxygen species and insulin resistance: the good, the bad and the ugly. Trends in pharmacological sciences. 2011;32(2):82–9. Epub 2010/12/17. doi: 10.1016/j.tips.2010.11.006 . 21159388 10.1016/j.tips.2010.11.006

[18] Erion DM, Shulman GI. Diacylglycerol-mediated insulin resistance. Nature medicine. 2010;16(4):400–2. Epub 2010/04/09. doi: 10.1038/nm0410-400 ; PubMed Central PMCID: PMC3730126. 20376053 10.1038/nm0410-400 PMC3730126

[19] Bays H, Mandarino L, DeFronzo RA. Role of the adipocyte, free fatty acids, and ectopic fat in pathogenesis of type 2 diabetes mellitus: peroxisomal proliferator-activated receptor agonists provide a rational therapeutic approach. The Journal of clinical endocrinology and metabolism. 2004;89(2):463–78. doi: 10.1210/jc.2003-030723 . 14764748 10.1210/jc.2003-030723

[20] Brewer PD, Habtemichael EN, Romenskaia I, Corley Mastick C, Coster AC. Insulin-Regulated Glut4 Translocation: Membrane Protein Trafficking With Six Distinctive Steps. The Journal of biological chemistry. 2014 Epub 2014/04/30. doi: 10.1074/jbc.M114.555714 . 24778187 10.1074/jbc.M114.555714 PMC4067164

[21] Ebbert JO, Jensen MD. Fat depots, free fatty acids, and dyslipidemia. Nutrients. 2013;5(2):498–508. doi: 10.3390/nu5020498 ; PubMed Central PMCID: PMC3635208. 23434905 10.3390/nu5020498 PMC3635208

[22] Wilcox G. Insulin and insulin resistance. The Clinical biochemist Reviews / Australian Association of Clinical Biochemists. 2005;26(2):19–39. ; PubMed Central PMCID: PMC1204764. 16278749 PMC1204764

[23] Morral N, Edenberg HJ, Witting SR, Altomonte J, Chu T, Brown M. Effects of glucose metabolism on the regulation of genes of fatty acid synthesis and triglyceride secretion in the liver. Journal of lipid research. 2007;48(7):1499–510. doi: 10.1194/jlr.M700090-JLR200 . 17449907 10.1194/jlr.M700090-JLR200

[24] Pacini G, Omar B, Ahren B. Methods and models for metabolic assessment in mice. Journal of diabetes research. 2013;2013:986906 doi: 10.1155/2013/986906 ; PubMed Central PMCID: PMC3673320. 23762879 10.1155/2013/986906 PMC3673320

[25] Khuwaja AK, Lalani S, Dhanani R, Azam IS, Rafique G, White F. Anxiety and depression among outpatients with type 2 diabetes: A multi-centre study of prevalence and associated factors. Diabetology & metabolic syndrome. 2010;2:72 doi: 10.1186/1758-5996-2-72 ; PubMed Central PMCID: PMC3022608. 21171976 10.1186/1758-5996-2-72 PMC3022608

[26] Jaworski K, Sarkadi-Nagy E, Duncan RE, Ahmadian M, Sul HS. Regulation of triglyceride metabolism. IV. Hormonal regulation of lipolysis in adipose tissue. American journal of physiology Gastrointestinal and liver physiology. 2007;293(1):G1–4. doi: 10.1152/ajpgi.00554.2006 ; PubMed Central PMCID: PMC2887286. 17218471 10.1152/ajpgi.00554.2006 PMC2887286

[27] Wu Y, Song P, Zhang WC, Liu JH, Dai XY, Liu ZY, et al Activation of AMPK alpha 2 in adipocytes is essential for nicotine-induced insulin resistance in vivo. Nature medicine. 2015;21(4):373–+. doi: 10.1038/nm.3826 WOS:000352493600019. 25799226 10.1038/nm.3826 PMC4390501

[28] Uchida Y, Takeshita K, Yamamoto K, Kikuchi R, Nakayama T, Nomura M, et al Stress augments insulin resistance and prothrombotic state: role of visceral adipose-derived monocyte chemoattractant protein-1. Diabetes. 2012;61(6):1552–61. doi: 10.2337/db11-0828 ; PubMed Central PMCID: PMC3357288. 22396205 10.2337/db11-0828 PMC3357288

[29] Gannouni N, Mhamdi A, Tebourbi O, El May M, Sakly M, Rhouma KB. Qualitative and quantitative assessment of noise at moderate intensities on extra-auditory system in adult rats. Noise & health. 2013;15(67):406–11. Epub 2013/11/16. doi: 10.4103/1463-1741.121236 . 24231419 10.4103/1463-1741.121236

[30] Gonzalez-Perez O, Chavez-Casillas O, Jauregui-Huerta F, Lopez-Virgen V, Guzman-Muniz J, Moy-Lopez N, et al Stress by noise produces differential effects on the proliferation rate of radial astrocytes and survival of neuroblasts in the adult subgranular zone. Neuroscience research. 2011;70(3):243–50. Epub 2011/04/26. doi: 10.1016/j.neures.2011.03.013 . 21514330 10.1016/j.neures.2011.03.013

[31] Manikandan S, Padma MK, Srikumar R, Jeya Parthasarathy N, Muthuvel A, Sheela Devi R. Effects of chronic noise stress on spatial memory of rats in relation to neuronal dendritic alteration and free radical-imbalance in hippocampus and medial prefrontal cortex. Neuroscience letters. 2006;399(1–2):17–22. Epub 2006/02/17. doi: 10.1016/j.neulet.2006.01.037 . 16481110 10.1016/j.neulet.2006.01.037

[32] Samson J, Sheeladevi R, Ravindran R, Senthilvelan M. Stress response in rat brain after different durations of noise exposure. Neuroscience research. 2007;57(1):143–7. Epub 2006/11/10. doi: 10.1016/j.neures.2006.09.019 . 17092591 10.1016/j.neures.2006.09.019

[33] Sjostrand M, Eriksson JW. Neuroendocrine mechanisms in insulin resistance. Molecular and cellular endocrinology. 2009;297(1–2):104–11. Epub 2008/07/05. doi: 10.1016/j.mce.2008.05.010 . 18599191 10.1016/j.mce.2008.05.010

[34] Taban E, Mortazavi SB, Vosoughi S, Khavanin A, Asilian H. Noise Exposure Effects on Blood Glucose, Cortisol and Weight Changes in the Male Mice. Health Scope. 2017;6(2). ARTN e36108 doi: 10.5812/jhealthscope.36108 WOS:000411529700001.

[35] Pankow JS, Duncan BB, Schmidt MI, Ballantyne CM, Couper DJ, Hoogeveen RC, et al Fasting plasma free fatty acids and risk of type 2 diabetes: the atherosclerosis risk in communities study. Diabetes care. 2004;27(1):77–82. . 14693970 10.2337/diacare.27.1.77

[36] Konkle ATM, Keith SE, McNamee JP, Michaud D. Chronic noise exposure in the spontaneously hypertensive rat. Noise & health. 2017;19(90):213–21. doi: 10.4103/nah.NAH_15_17 ; PubMed Central PMCID: PMC5644380. 28937015 10.4103/nah.NAH_15_17 PMC5644380

[37] Armario A, Montero JL, Pla-Giribert T, Vivas C, Balasch J. Effect of chronic noise or water restriction on weight of body and organs in the rat. Revista espanola de fisiologia. 1983;39(3):267–70. . 6658142

[38] Garza JC, Guo M, Zhang W, Lu XY. Leptin restores adult hippocampal neurogenesis in a chronic unpredictable stress model of depression and reverses glucocorticoid-induced inhibition of GSK-3beta/beta-catenin signaling. Molecular psychiatry. 2012;17(8):790–808. doi: 10.1038/mp.2011.161 ; PubMed Central PMCID: PMC3368076. 22182938 10.1038/mp.2011.161 PMC3368076

